# Does influenza vaccination help reduce incidence of COVID-19 infection among hospital employees?

**DOI:** 10.1097/MD.0000000000028479

**Published:** 2022-01-14

**Authors:** Soha H. Shosha, Dana I. Ajlan, Rana Al-Ghatam

**Affiliations:** Primary Health Care Department, Bahrain Defense Force Hospital, Rifaa, Bahrain.

**Keywords:** COVID-19, healthcare workers, influenza vaccine

## Abstract

To facilitate the understanding of the interaction between severe acute respiratory syndrome coronavirus 2 causing the corona virus disease 2019 (COVID-19) and other pathogens causing respiratory system affection we investigated the effect of influenza vaccination on the incidence and severity of COVID-19 among members of staff working in the Bahrain Defense Force Hospital.

All staff members working in the hospital between February 2020 and March 2021 were divided into 2 main groups based on whether or not they received influenza vaccination. None of the participants had received any of the COVID-19 vaccines throughout this time period. The records of each were scrutinized to see the effect of influenza vaccination on incidence and severity of COVID-19. Severity measures were: need for hospital and intensive care unit admission and total length of hospital stay.

Incidence of affection with COVID-19 was much lower in the vaccinated group (3.7% vs 8.1%, *P* < .001). Influenza vaccination also reduced total length of hospital stay (6.2 days vs 12.7 days, *P* < .05) and need for intensive care unit admission among the patients.

Influenza vaccine reduces both the incidence of affection as well as the overall burden of COVID-19. This is of particular importance for people working in the healthcare field during the serious COVID-19 pandemic.

## 1 Introduction

Corona virus disease 2019 (COVID-19) was declared as a pandemic in March 2020.^[1]^ It is caused by a pathogen named “severe acute respiratory syndrome coronavirus 2” (SARS-CoV-2).^[2]^ Infection by COVID-19 causes pneumonia in many cases which can be fatal especially among vulnerable age groups and patients with pre-existing conditions.^[3]^ The risk of severe COVID-19 will probably also increase if there is co-infection with other pathogens such as influenza viruses because they also cause inflammation of the respiratory system.^[4–6]^ Seasonal influenza vaccines help reduce the risk of becoming infected with many of the viruses that cause respiratory tract infection^[7]^ as well as reducing the overall severity of the disease.^[8]^ Intuitively, by reducing the risk of infection, influenza vaccination has the potential to minimize the number of influenza related hospital admissions as well as COVID-19 related morbidity. This will ultimately help reduce the resources needed to care for both COVID-19 and non-COVID-19 patients during the pandemic.^[9]^ Furthermore, because of the high degree of overlap between the symptoms of influenza and COVID-19, vaccination will also probably increase the specificity of syndromic surveillance and the overall reporting of the burden of the disease.^[9]^ This is why it is of paramount importance to investigate the usefulness of influenza vaccination during the COVID-19 pandemic. Subjects working in healthcare interact with infected patients on a regular basis and therefore are at high risk of acquiring all forms of transmittable diseases including seasonal influenza as well as COVID-19. Vaccination of those groups will potentially reduce the risk of healthcare acquired COVID-19 infection or at least minimize associated morbidity due to co-infecting pathogens.^[10]^ Delineating the effect of influenza vaccination is not only important for the healthcare workers but also the risk on their families and the community as a whole.

In the current study we ask whether vaccination with seasonal influenza vaccine helps reduce the incidence of COVID-19 viral infection and morbidity in the employees working at the Bahrain Defense Force Hospital (BDF) during the first year of the COVID-19 pandemic, prior to the beginning of mass vaccination with the COVID-19 vaccine.

## 2 Methods

### 2.1 Study design and participants

This is a retrospective observational study carried out on the employees working at the BDF between February 21, 2020 and March 31, 2021. This is the period of time that started after the detection of the first COVID-19 case in Bahrain^[11]^ and ended as soon as mass immunization with COVID-19 vaccine started in our hospital. The BDF is one of the largest hospitals in Bahrain with over 3000 employees providing healthcare related services.

The participants were divided into 2 main groups; **Vaccinated Group:** those who received seasonal influenza vaccination and **Non-Vaccinated Group:** those who did not receive this vaccine. Each of those main groups were then sub-divided based on whether they were infected with the COVID-19 virus or not.

The vaccinated subjects received the influenza vaccine either during the October 2019 or the January 2020 influenza vaccination campaign conducted by the hospital.

**Inclusion criteria:** All employees providing any kind of service at the hospital. This encompassed medical staff; doctors (including dentists) and nurses, paramedical personnel; psychologists, paramedics, radiologists, technicians, physiotherapists, dietitians, speech therapists respiratory care therapists, optometrists, pharmacists, and social workers; clerical and office staff; management, receptionists, secretaries, IT specialists, and accountants; and supportive jobs; janitors, cleaners, security guards, maintenance workers, kitchen personnel, drivers, store keepers, etc.

**Exclusion criteria:** receiving COVID-19 vaccine at any time during the study period, any one diagnosed with COVID-19 prior to the study period, and for the vaccinated group; subjects who contracted COVID-19 less than 2 weeks after receiving the vaccine or over 12 months after vaccination (duration of effectivity of the influenza vaccine).^[12]^

The study was approved by the local ethics committee (registration and approval number CRT-COVID2021-121). Consent was waived by the ethics committee since the study involved the analysis of the existing personnel information after removal of the name and employee number thus did not interfere in any way with their management or confidentiality.

### 2.2 Data collection and statistical analysis

The records of every member of staff working at the hospital were first anonymized then scrutinized to see if and when they received the influenza vaccine and whether any of them was diagnosed with COVID-19 during the study period.

The data were then analyzed according to:

1) Demographic factors:
  a. Age.
  b. Gender.
  c. Job category:
    i. Medical staff.
    ii. Paramedical personnel.
    iii. Employees performing clerical and office work.
    iv. Members of staff performing supportive jobs.
  d. Pre-existing chronic medical conditions if present:
    i. Diabetes.
    ii. Cerebrovascular disorders including hypertension and ischemic heart disease.
    iii. Chest diseases including allergies, asthma, and chronic obstructive pulmonary disease.
    iv. Neurological conditions including migraine, immune mediated such as multiple sclerosis, neuropathies, and radiculopathies.
2) Patients who developed COVID-19:
  a. Time interval between vaccination and infection (vaccinated group only).
  b. Disease severity:
    i. Management at home.
    ii. Hospitalized.
    iii. Admission to the intensive care unit (ICU).
    iv. Deceased.
  c. Total length of hospital stay in days for those hospitalized and/or admitted to the ICU.

### 2.3 Data analysis

Descriptive analysis was done using SPSS version 26 (IBM SPSS Statistics for Windows, Armonk, NY) which computed simple descriptive analysis including frequencies for categorical data, mean & standard deviation for numerical data. Cross tabulation was done for some of the categorical data using Chi-Square, Fisher Exact, and Cramer V to test when there was a statistically significant difference between the groups. Correlations between quantitative variables were done using Spearman correlation coefficient. 95% confidence interval was computed for all independent variables. *P* values less than .05 were considered as statistically significant.

## 3 Results

### 3.1 Demographic data

**Figure 1:**
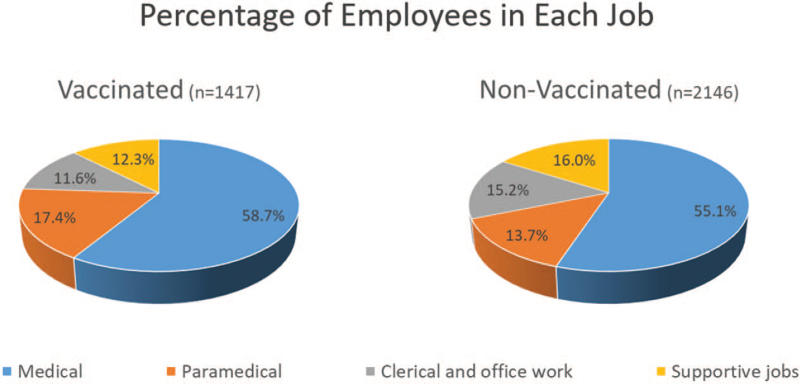
Distribution of staff members in each job category among the vaccinated and non-vaccinated groups.

There were 3566 members of staff working at the BDF during the study period time. A total of 1420 employees had received the seasonal influenza vaccine (mean age 39.31 ± 8.78, range 21–64 years old). Only 1417 were included in the study (514 males and 903 females). The remaining 3 were excluded because they became COVID-19 positive less than 5 days after receiving the influenza vaccine (immunity starts 2 weeks after vaccination^[12]^). The non-vaccinated group contained 2146 employees (mean age 41.84 ± 11.6, range 20–65 years, 830 males and 1316 females). There were no significant differences in these variables between the 2 groups. There was also no difference in the number of subjects employed under each job between the 2 groups (Fig. 1). It was notable however that the number of staff who did not receive the influenza vaccine was almost double those who did.

**Figure 2:**
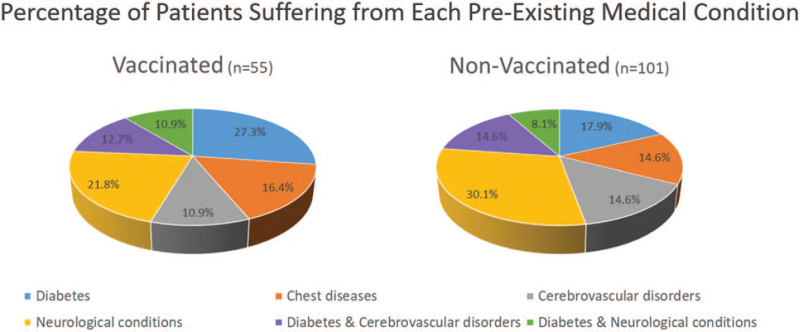
Prevalence of pre-existing medical conditions among the members of staff who suffer from each condition in the vaccinated and non-vaccinated groups.

Pre-existing medical conditions were found in 55 (3.9%) employees in the vaccinated group, and 101 (4.7%) members in the non-vaccinated group. The prevalence of these conditions in each group is displayed in Figure 2. Of note, migraine (categorized under neurological conditions) was the most prevalent followed by diabetes and then hypertension (categorized under cardiovascular disorders). There were no differences in the prevalence of any of the conditions between the 2 groups (vaccinated vs non-vaccinated).

### 3.2 COVID-19 infection

#### 3.2.1 Incidence

The first case of COVID-19 among our hospital staff was recorded on the April 14, 2020. She was a nurse and was in the non-vaccinated group. The first case in the vaccinated group was also a nurse and she presented a month later (May 13, 2020). Infection occurred between 14 and 350 days after vaccination (mean: 145.52 ± 103.58).

**Figure 3:**
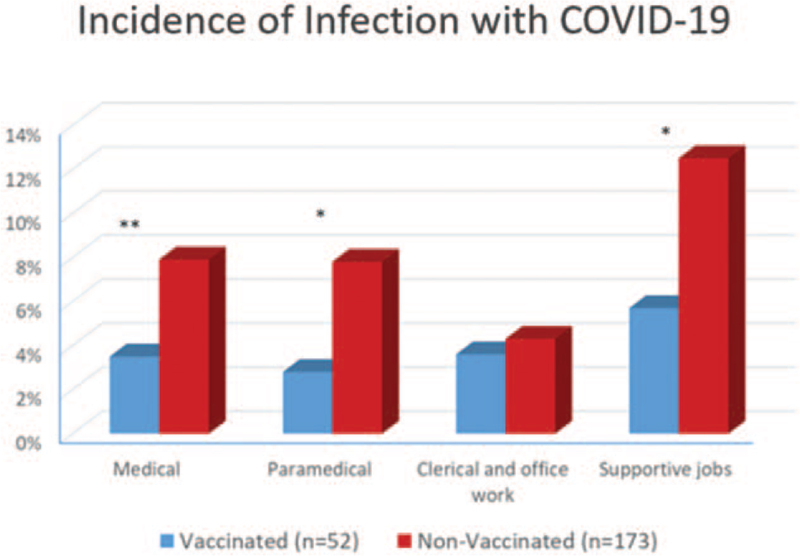
Incidence of infection with COVID-19 among the members of staff who got infected classified according to job in the vaccinated and non-vaccinated groups. ^∗∗^ = *P* < .01 and ^∗^ = *P* < .05. COVID-19 = corona virus disease 2019.

The incidence of infection with COVID-19 was 3.7% (52 patients) in the vaccinated group. This was significantly lower compared to the non-vaccinated group where the incidence was 8.1% (173 patients; *P* < .001). As shown in Figure 3, the highest rate of infection was among the staff members performing supportive jobs in both groups, however there was no significant correlation between incidence of infection and the job description in the vaccinated or non-vaccinated group. There was also no significant correlation between the incidence of infection and age, gender, or presence of pre-existing medical conditions in either group.

On comparison between the 2 groups (vaccinated and non-vaccinated), the difference in incidence of infection was most marked in the medical (*P* ≤ .001) and paramedical staff (*P* = .012) followed by the staff performing supportive jobs (*P* = .016). It failed to reach significance in the staff members doing office related work (*P* = .72).

#### 3.2.2 Severity

**Figure 4:**
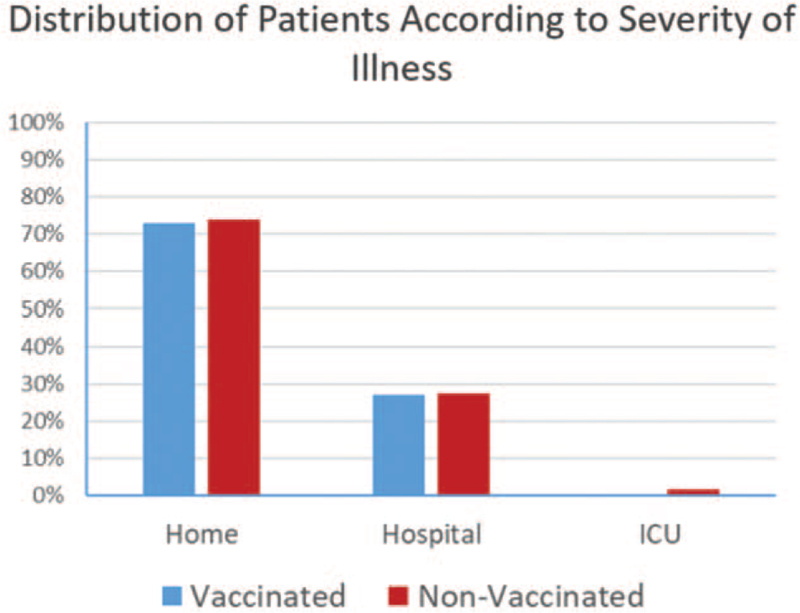
Distribution of patients according to severity of illness and need for hospitalization and ICU admission in the vaccinated and non-vaccinated groups. ICU = intensive care unit.

Most of the patients in both groups presented with mild symptoms and were treated at home (Fig. 4). There was no significant difference between the patients who needed to be hospitalized in the vaccinated group compared to the non-vaccinated group though the 3 who suffered from severe symptoms that required admission to the ICU were all in the non-vaccinated group. All the patients recovered and there were no fatalities in either group (vaccinated or non-vaccinated). All job categories showed similar findings with no differences between them in either group. There was also no correlation between hospitalization or admission to the ICU and presence of pre-existing medical conditions.

**Figure 5:**
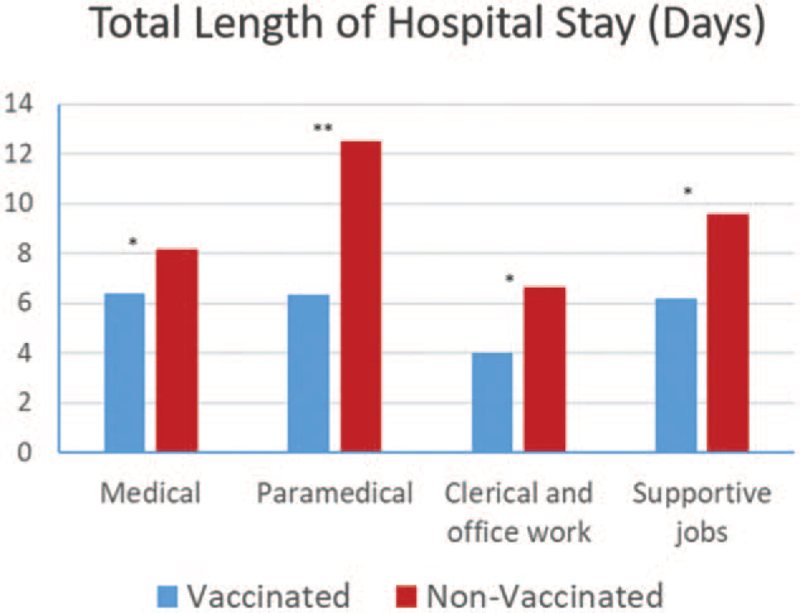
Total length of stay in the hospital in days in each job category among the vaccinated and non-vaccinated group. ^∗∗^ = *P* < .01 and ^∗^ = *P* < .05.

The total length of hospital stay in days was significantly lower in the vaccinated group (6.2 ± 3.8; range 3–10 days) compared to the non-vaccinated group (12.7 ± 6.9; range 3–21 days, *P* = .038). As illustrated in Figure 5, this was noted in all job categories and was most marked in the paramedical personnel. It was not affected by age or gender however it correlated positively with pre-existing medical conditions in a similar fashion in both the vaccinated and non-vaccinated groups (*P* = .042 and *P* = .048, respectively) with no difference between them. Even though the longest length of hospital stay was observed in the patients with cardiovascular disorders whether they were vaccinated or not, this failed to reach significance and there were no differences between the different categories of illnesses in either group.

There was a significant positive correlation between the time after vaccination to infection and the total length of hospital stay in days (*P* = .012). The time between vaccination and infection was also higher in the patients who were hospitalized or admitted to the ICU (mean 184.47 ± 113.65) compared to those who remained at home (mean 126.6 ± 84.78) however this difference failed to reach significance (*P* = .058).

## 4 Discussion

To our knowledge, this is the first study investigating the effect of seasonal influenza vaccination on incidence and severity of COVID-19 on members of staff working in a hospital. The COVID-19 pandemic has had dire consequences on global health and the economy. Despite the huge number of infected people worldwide, there are still many unanswered questions relating to COVID-19 morbidity and risk of infection. Studying the effect of influenza vaccination on the members of staff working in a facility providing healthcare is an ideal setting because they are the cohorts most exposed to both forms of infection. Furthermore, working in hospitals encompasses all levels of exposure to patients ranging from the medical staff who are at very high risk of getting infected to employees with minimal contact with patients such as store keepers and members of staff performing pure office and administrative jobs.

The total number of cases with COVID-19 recorded in Bahrain during the study period (February 21, 2020–March 31, 2021) was 143,574 (incidence of infection 8.4%) with 523 deaths.^[13,14]^ We observed a similar incidence (8.1%) of infection in the non-vaccinated group. The notable finding in our study was the much lower incidence of infection (3.7%) in the group who received influenza vaccination. We were expecting the incidence to be highest among the medical and paramedical personnel, however it was highest in the employees performing supportive jobs in both the vaccinated and non-vaccinated groups. Perhaps this can be explained by the fact that this group included janitors and cleaners. As with medical and paramedical personnel those staff members also have close contact with patients on a daily basis and are thus at high risk of infection. As expected, the incidence was lowest in the members of staff with pure office work and minimal contact with patients.

The interaction of SARS-CoV-2 with other pathogens including influenza viruses remains unknown. Both SARS-CoV-2 and influenza viruses are single-stranded enveloped RNA viruses.^[15]^ SARS-CoV-2 is a positive-sense single-stranded RNA virus while influenza viruses are negative-sense single-stranded RNA viruses that have 4 types: A, B, C, and D. Types A and B incorporate many clades and subclades based on surface proteins and genetic properties. Seasonal flu epidemics are mainly caused by types A and B.^[15]^ For type A the most virulent subtypes are HA (H1, H2, and H3), and NA (N1 and N2) while for type B the 2 most well-known lineages: B/Yamagata/16/88-like, and B/Victoria, are the main culprits but they have a lower impact than type A.^[16,17]^ Both SARS-CoV-2 and influenza viruses can attack the respiratory system of humans producing similar symptoms including fever, cough, and shortness of breath.^[18]^ Influenza viruses causes seasonal flu epidemics each year while SARS-CoV-2 runs a far less predictable course, is much more easily transmissible, and is not seasonal, as shown by the ever-increasing numbers of infection throughout the year with no clear association with the weather.^[19]^

Many forms of influenza vaccines exist. In our hospital we use the quadrivalent vaccine which contains 2 A; A(H1N1) and A(H3N2) and 2 influenza B live attenuated viruses. Little is known about how influenza vaccination interacts with corona viruses. In a recent report emerging months after the onset of the pandemic year the authors found that receipt of influenza vaccine was associated with greater risk of 4 types of coronaviruses including SARS-CoV-2.^[20]^ This would’ve had profound repercussions on subsequent use of influenza vaccine during the current pandemic if these findings had not been quickly negated by a much larger study that showed that the influenza vaccine significantly reduced the risk of influenza illness by >40% with no effect on coronaviruses or other non-influenza respiratory viruses.^[20]^ In particular, influenza vaccine did not affect seasonal coronavirus risk.^[20,21]^ This is corroborated by the findings in our study and provides substantial reassurance against the view that influenza vaccine may negatively affect COVID-19 risk.

Most of the patients who became ill with COVID-19 in our study suffered from mild symptoms that did not require hospitalization and there were no fatalities in either group. The important finding though was that even though the need for hospitalization was similar in both groups, patients in the non-vaccinated group were hospitalized for a significantly longer period (range of 3–10 days in the vaccinated group vs 3–21 days in the non-vaccinated group). In addition, ICU admission was only found in the non-vaccinated group. Furthermore, the total length of hospital stay was significantly longer with increased time interval between influenza vaccination and infection with COVID-19. This suggests that influenza vaccination reduces the likelihood of occurrence of COVID-19 related complications. Most reports show that the symptoms in COVID-19 are usually self-limiting mild-to-moderate influenza-like and only a small group of patients have significant problems that require hospitalization or admission to the ICU.^[3,22]^ These complications can involve multiple systems causing severe acute cardiovascular and cardiopulmonary complications such as multiple organ failure, shock, acute respiratory distress syndrome, myocarditis, heart failure, and arrhythmias as well as multiple autoimmune reactions. These significant detriments can be fatal especially in high-risk groups. The population cohorts most susceptible for developing these impairments are the elderly^[3,23]^ and those with pre-existing co-morbidities such as diabetes, hypertension, and ischemic heart diseases.^[24–28]^ These high-risk populations are shared by both COVID-19 and seasonal influenza^[23]^ however, COVID-19 is much more deadly and is associated with a 10 time higher hospitalization rate and a 5 to 10 times higher mortality rate.^[18,29,30]^ In our opinion one of the strengths of our study design is that it only included relatively healthy adults who are in the work force. The oldest participant in both groups was 65 years old and there was a very low prevalence of pre-existing medical conditions (3.7% in the vaccinated group and 4.7% in the non-vaccinated group). While presence of pre-existing conditions especially cardiovascular disorders was associated with a slightly longer length of hospital stay, the numbers of patients who suffered from these conditions are too small which probably downgrades the potential confounding effect of these illness on the findings. Absence of subjects over the age of 65 years also overcomes the problematic possible reduced efficacy of the vaccine in older populations.^[12]^ This facilitates a more objective appraisal of the actual effect of influenza vaccine on COVID-19. It is quite likely that co-infection with both SARS-CoV-2 and influenza virus results in a more severe course, a higher likely hood of complications and/or fatality.^[31,32]^ Our findings suggest that influenza vaccination can reduce the possibility of such dire consequences. Research has shown that antibodies start to develop in the body about 2 weeks after influenza vaccination and immunity lasts for up to 12 months thereafter. While this is not 100% effective in preventing infection, it has been shown to significantly reduce the severity of illness in those who do get sick including reduction of number of fatalities, ICU admissions, duration of stay in the ICU, and the overall period of hospitalization in those who required hospitalization.^[3,33]^ A number of hypotheses have been proposed to explain why this happens. Influenza vaccination reduces the risk of infection and associated pathological consequences which is important in itself, however, the vaccine itself may interact with immune and inflammatory systems which helps plaque stabilization.^[34,35]^ Furthermore, the antibodies caused by the vaccine may also interact with the bradykinin 2 receptor, leading to increased nitric oxide production.^[34,36]^ This may significantly reduce the probability of development of severe complications especially in high-risk cardiovascular patients and may even offer some incremental cardiorespiratory protection.^[34]^

Long term follow up after recovery was not part of our study design which is probably the main limitation of the current study. There is now growing evidence that there are post-COVID conditions that incorporate a wide range of new, returning, or ongoing health problems that start more than 4 weeks after first being infected with SARS-CoV-2 even in patients who were symptom free or had mild symptoms.^[34]^ We aim to investigate this further in the near future.

## 5 Conclusion

The findings in the present study provide evidence that influenza vaccination decreases the incidence of infection with COVID-19 as well as the associated morbidity. We believe these are important findings especially since they were obtained from a large cohort of healthcare personnel.

It is likely that influenza vaccination would be considered as a fundamental part of the management plans for the COVID-19 pandemic, however more immune-epidemiological studies are probably needed to confirm this. These will need to include other age groups since only adults were included in the current study and the most vulnerable cohorts are the elderly. More work is also needed in the pediatric age groups. Further validation would also require delineating the effect of co-vaccination with both influenza and COVID-19 vaccines on the risk of infection and development of complications. This is the logical next step and is quite feasible now that mass immunization with different types of COVID-19 vaccine programs is being implemented worldwide. In our view, this would provide very important information that will help reduce the various complications and occupational hazards and consequently the cost and burden of management of patients during the current pandemic.

## Acknowledgments

We wish to show appreciation to the Supreme Council of Health and Major General/Professor Khalid bin Ali Al Khalifa the Director of Royal Medical Services at the Bahrain Defense Force Hospital for authorization to conduct this research. Special thanks goes to the Head of the IT department Dr Muzammil for his assistance with data entry and the statisticians for their help with data analysis. Finally, we are grateful for the support of all the members of staff who helped and encouraged us during this research.

## Author contributions

All authors participated in study design, data collection and analysis, and manuscript preparation.

**Conceptualization:** Soha Shosha, Dana I Ajlan, Rana Al-Ghatam.

**Data curation:** Soha Shosha.

**Formal analysis:** Soha Shosha, Rana Al-Ghatam.

**Funding acquisition:** Soha Shosha, Rana Al-Ghatam.

**Investigation:** Soha Shosha, Dana I Ajlan.

**Methodology:** Soha Shosha, Dana I Ajlan, Rana Al-Ghatam.

**Project administration:** Soha Shosha, Dana I Ajlan.

**Resources:** Soha Shosha, Dana I Ajlan.

**Software:** Soha Shosha.

**Supervision:** Rana Al-Ghatam.

**Validation:** Soha Shosha, Dana I Ajlan.

**Visualization:** Soha Shosha.

**Writing – original draft:** Soha Shosha, Dana I Ajlan, Rana Al-Ghatam.

**Writing – review & editing:** Soha Shosha, Dana I Ajlan, Rana Al-Ghatam.
